# Differences in Statin Usage and Target-Goal Achievement between Departments at the Same Hospital

**DOI:** 10.1371/journal.pone.0050466

**Published:** 2012-12-10

**Authors:** Zhong Chen, Xin Wang, Zhen Ding, Peiying Fan, Genshan Ma

**Affiliations:** Department of Cardiology, The Affiliated Zhongda Hospital and School of Medicine, Southeast University, Nanjing, P. R. China; Medical University Innsbruck, Austria

## Abstract

**Objective:**

To compare use of statins and target-goal achievement in patients with type 2 diabetes mellitus (T2DM), with or without stable coronary artery disease (CAD), between cardiology and endocrinology departments at a tertiary hospital.

**Methods:**

A total of 966 patients with T2DM were enrolled, including 553 with stable CAD, from the departments of endocrinology and cardiology. Baseline characteristics, prescription of statins, and target-goal achievement of low-density lipoprotein cholesterol (LDL-C) during a 6-month follow-up period were analyzed.

**Results:**

There was lower ratio of statin use in patients with T2DM, with or without CAD, in the department of endocrinology than in the department of cardiology (all *P*<0.05). At the 6-month follow-up, compared to patients with T2DM in the endocrinology department, target-goal achievement among patients with T2DM in the department of cardiology was higher (52.90% vs. 41.46%, P<0.01), indicating a significant improvement among patients in the department of cardiology but not for those in the department of endocrinology when compared to baseline. According to the new Chinese guidelines, the goal attainment rate was higher among patients with T2DM combined with CAD in the department of cardiology than in the department of endocrinology (27.62% vs. 19.05%, *P*<0.05). However, with regard to ATP III 2004, the goal attainment rate was similar for patients with T2DM combined with CAD in both departments during the 6-month follow-up (9.21% vs. 8.84%, *P*>0.05), with no apparent improvement compared to baseline.

**Conclusions:**

There was differential and sub-optimal use of statins as well as low target-goal achievement among patients with T2DM, with or without CAD, in the departments of cardiology and endocrinology at the same tertiary hospital, with a lower rate of statin prescription and target-goal achievement of LDL-C in the department of endocrinology.

## Introduction

Coronary artery disease (CAD) is one of the major causes of death in most countries, including China [1]. More importantly, the prevalence of obesity and type 2 diabetes mellitus (T2DM) is increasing rapidly in China, as less than 1% of Chinese adults had T2DM in 1980 and this had risen to nearly 10% by 2008 [2], [3]. Patients with T2DM have an increased incidence of atherosclerotic cardiovascular disease, and patients with CAD and T2DM belong to a very-high-risk population [4]–[11], which deserves more attention from doctors and health professionals.

Fortunately, numerous evidence-based interventions exist, ranging from glycemic and cardiometabolic risk-factor control to early screening for diabetes complications [12], and great progress has been achieved, as evidenced in population-based studies [13]–[15]. Among these interventions, the protective effects of cholesterol-lowering therapy with statins in primary and secondary prevention have been well verified and acknowledged by many guidelines, and low-density lipoprotein cholesterol (LDL-C) control is the first target goal [8]–[11], [16]–[21].

Despite these improvements, differences remain between Chinese and Western populations in regard to the control of cardiovascular disease (CVD) risk factors, especially in regard to target-goal achievement for LDL-C [22]–[24]. Study results from the U.S. population reveal that death rates among both U.S. men and women with diabetes declined substantially between 1997 and 2006, along with a reduction in the absolute difference between adults with and without diabetes [25]. Aggressive application of nationally recommended prevention activities for CVD would potentially add millions of quality adjusted life-years to the adult population in the U.S. and improve the average lifespan by at least 1.3 years [26].

Trials specifically performed in subjects with T2DM have consistently demonstrated significant benefits of statin therapy on CVD events in people with T2DM [27]. Patients with CAD and T2DM need to achieve a more strict target-goal value [8]–[11]. It is still unknown whether there exist differences in statin usage and target-goal achievement in T2DM patients with or without stable CAD between different departments at the same tertiary hospitals, besides great progress having been made in this field. To fill this knowledge gap, we aimed to determine whether differences existed in statin usage and LDL-C target-goal attainment in T2DM patients with or without stable CAD between the cardiology and endocrinology departments at the same tertiary hospital, mainly including Chinese Han patients living in Southeast China. The results of this study will help understand these potential differences and provide new data for reference in decision-making aimed at reducing long-term cardiovascular risks.

## Methods

### Study population samples

This study is a cross-sectional survey and midterm follow-up study. From January 2008 to December 2009, 966 patients diagnosed with T2DM were enrolled in this study, including 553 patients with documented stable CAD, from the departments of cardiology and endocrinology of the Affiliated Zhongda Hospital of Southeast University, a tertiary hospital located in Nanjing, Jiangsu Province, China. CAD was defined as a significant coronary stenosis (≥50%) in at least one of the three main coronary arteries or their major branches (branch diameter ≥2 mm) as assessed by coronary angiography (CAG) or having experienced a myocardial infarction defined according to World Health Organization criteria. All patients with type 1 diabetes, acute coronary syndrome, a history (within the past month) of acute infection requiring antibiotic therapy, a recent (within the past month) or abrupt change in their usual diet; pregnant women or women who were breastfeeding, congenital heart disease, syndrome X, severe liver or kidney disease, noncoronary artery thrombotic disease, or any known secondary cause of dyslipidemia were excluded from this study. The study was approved by the Medical Ethics Committee of the Affiliated Zhongda Hospital of Southeast University. Before enrollment, the trial information was explained carefully to each patient, and subsequent written informed consent was obtained from all participants.

### Coronary angiography and percutaneous coronary intervention

All participants had records of CAG during hospitalization or within one year. The grade of the coronary stenosis and CAD diagnosis were judged by two cardiologists that were unaware of this study. Percutaneous coronary intervention (PCI) was done at the discretion of the cardiologists under the current guidelines.

### Determination of parameters and risk factors

At the time of enrollment, interviewers assessed diabetes status by asking each participant if a doctor had ever told her/him that he/she had diabetes. In addition, respondents were queried for age, gender, and risk factors including hypertension, T2DM, smoking status, and family history of CVD. Anthropometric measurements and blood pressure determination were performed according to standard protocols. Fasting blood samples were collected and plasma concentrations of total cholesterol (TC), triglycerides (TG), high-density lipoprotein cholesterol (HDL-C), and LDL-C were analyzed as previously described [28]. Levels of apolipoprotein A1 (apo A1) and apolipoprotein B (apo B) were determined by standard biochemical methods using a chemistry analyzer (Synchron clinical system LX20; Beckman Coulter, Brea, CA, USA). Determination of hypertension, T2DM, and smoking status has been previously described [29]. Patients with a history of T2DM, those receiving antidiabetic medications, and those with confirmed fasting blood sugar >126 mg/dl (7.0 mmol/l) were considered to have T2DM. The physicians determined the prescription of lipid-lowering drugs at the hospital, and use of statins was recorded at discharge.

### Follow-up

All patients were followed up after discharge at an average interval of six months by the researchers without any extra intervention. The 6-month follow-up data was obtained by the investigators from all patients via regularly scheduled office visits. Twenty-seven patients dropped out during the 6-month follow-up and the follow up rate is 97.20%. Blood samples were collected after at least 8 h of fasting, and plasma was stored at −40°C for future LDL-C detection. All samples were thawed only once.

### Statistical analyses

Statistical analyses were conducted using SPSS 15.0 software (SPSS, Chicago, IL, USA). Data from LDL-C was normally distributed and presented as mean±SD, comparisons were analyzed using Student t-test; whereas skewed data including TC, TG, HDL-C, apo A1 and apo B were expressed as median and quartile ranges, and the comparisons were done after logistic transformations. Categorical variables were analyzed using the chi-square test. The goal attainment rate of LDL-C was defined as the percentage of patients reaching cholesterol goals recommended by the updated ATP III (Adult Treatment Panel III) of the National Cholesterol Education Program [8], [9] and the new Chinese Guidelines on Prevention and Treatment of Dyslipidemia in Adults [10]. Generally speaking, the study design consists of 2 parallel cohorts (1. patients with T2DM, and 2. patients with T2DM and stable CAD) from cardiology and endocrinology departments and they are evaluated separately. Two-tailed *P* values*<*0.05 were considered significant.

## Results

### Analysis of baseline characteristics in patients from the two departments (Table 1)

A total of 966 patients with T2DM were enrolled, among which 562 were from the department of cardiology and 404 from the department of endocrinology. Table 1 summarizes the basic characteristics of the study population. At baseline, patients with T2DM from the department of endocrinology were younger than those from cardiology or compared to patients with T2DM combined with CAD in endocrinology (*P*<0.01). There was a lower ratio of statin use among patients with T2DM with or without CAD and a lower ratio of patients undergoing percutaneous coronary intervention among the patients with T2DM and CAD in the department of endocrinology than among those in cardiology (all *P*<0.05). At baseline, patients with T2DM or combined with CAD in both the endocrinology and cardiology departments were comparable for levels of LDL- C, TC, TG, apo A1, apo B, and ratio of apo B/apo A1 as well as gender, smoking, and hypertension (all *P*>0.05).

**Table 1:** Baseline characteristics of patients in the departments of cardiology and endocrinology.

Variables	T2DM		T2DM and stable coronary artery disease	
	Department of cardiology (n = 160)	Department of endocrinology (n = 253)	Department of cardiology (n = 402)	Department of endocrinology (n = 151)
Age, years (mean ± SD)	68.58±11.06	61.37±13.68†	69.48±9.81	68.95±10.10
Men, n (%)	89(55.60)	137(54.20)	208(51.70)	80(53.00)
Smoking, n (%)	36(22.50)	59(23.32)	92(22.89)	37(24.50)
Hypertension, n (%)	110(68.75)	168(66.40)	324(80.60)	119(78.80)
LDL-C, mmol/L	2.62±0.89	2.67±1.03	2.72±1.16	2.82±1.23
<1.8 (<70 mg/dl) (%)	--	--	27(6.71)	12(7.95)
<2.07 (<80 mg/dl) (%)	--	--	69(17.16)	25(16.57)
<2.60 (<100 mg/dl) (%)	58(36.25)	94(37.15)	--	--
Total cholesterol, mmol/L	4.02(3.79–5.25)	3.98(3.74–5.40)	4.10(3.90–5.32)	4.11(3.94–5.45)
Triglycerides, mmol/L	1.58(1.28–2.10)	1.54(1.37–2.23)	1.78(1.32–2.31)	1.74(1.40–2.39)
HDL-C, mmol/L	1.17(1.05–1.31)	1.10(0.94–1.29)	1.01(0.93–1.20)	1.03(0.90–1.24)
apo A1, g/L	0.88(0.76–1.02)	0.91(0.80–1.18)	0.90(0.83–1.21)	0.91(0.82–1.19)
apo B, g/L	0.73(0.70–0.86)	0.74(0.71–0.89)	0.76(0.70–0.86)	0.78(0.71–0.91)
apo B/apo A1	0.80±0.26	0.84±0.27	0.78±0.23	0.80±0.27
Statins, n(%)	103(64.38)	91(36.00)†	352 (87.56)	87(57.62)†
percutaneous coronary intervention, n (%)	--	--	284(70.65)	91(60.26)*

* *P*<0.05,

† *P*<0.01 compared with T2DM with or without stable coronary artery disease, respectively, in the department of cardiology.

### The results of the 6-month follow-up for the patients in the two departments (Table 1 and Table 2)

At the 6-month follow-up, compared to patients with T2DM in the department of endocrinology, the ratio of target-goal achievement of LDL-C (<100 mg/dl) among patients in the department of cardiology was higher (52.90% vs. 41.46%, *P*<0.01), indicating a significant improvement for patients in cardiology but not in endocrinology when compared to baseline (36.25% and 37.15%, respectively). According to the new Chinese guidelines, the goal attainment rate (LDL-C <80 mg/dl) was also higher among patients with T2DM and CAD in cardiology than in endocrinology (27.62% vs. 19.05%, *P*<0.05). However, with regard to ATP III 2004, the goal attainment rate (LDL-C<70 mg/dl) was similar in patients with T2DM and CAD from both departments (9.21% vs. 8.84%, *P*>0.05), without any significant improvement when compared to baseline (6.71% and 7.95%, respectively).

**Table 2:** Six-month follow-up results for patients in the departments of cardiology and endocrinology.

Variables	T2DM		T2DM and stable coronary artery disease	
	Department of cardiology (n = 155)	Department of endocrinology (n = 246)	Department of cardiology (n = 391)	Department of endocrinology (n = 147)
Statins, n (%)	99(63.87)	77(31.30)†	341(87.21)	75(51.02)*
LDL-C, mmol/L	2.21±0.84	2.58±0.97*	2.27±0.96	2.53±0.98*
<1.8 (<70 mg/dl) (%)	--	--	36(9.21)	13(8.84)
<2.07 (<80 mg/dl) (%)	--	--	108(27.62)	28(19.05)*
<2.60 (<100 mg/dl) (%)	82(52.90)	102(41.46)†	--	--

* *P*<0.05,

† *P*<0.01 compared with T2DM with or without stable coronary artery disease in cardiology.

## Discussion

To our knowledge, this study represents the first such report regarding statin use and LDL-C target-goal attainment in a sample of patients from China. We found sub-optimal use of statins and low target-goal achievement of LDL-C in patients with T2DM (combined with stable CAD or not) in both the departments of cardiology and endocrinology, with a lower rate of statin prescription and target-goal achievement of LDL-C in the department of endocrinology.

In Daqing, China, it has been reported that the frequency of CAD has markedly increased in patients newly diagnosed with diabetes than in patients without diabetes over a 20 year period [30]. Aggressive lipid management has recently become the standard of care for patients with CAD. However, until recently, the safety and effectiveness of statin usage for patients with extremely low LDL-C levels has been questioned. In the study by Leeper NJ et al. [31], a total of 6107 consecutive patients with LDL-C levels less than 60 mg/dL were identified from a tertiary care medical center or affiliated community clinic. Their mean age was 65 years, 43% had prior ischemic heart disease, and 47% had T2DM. During a mean follow-up of 2.0±1.4 years, statin therapy in the setting of a very low LDL-C level (<40–60 mg/dL) appeared to be safe and was associated with improved survival. Furthermore, statin use was not associated with an increase in malignancy, transaminase elevation, or rhabdomyolysis [31].

With a limited amount of available data, this is the first study done in a Chinese population analyzing the differences of drug-adherence and goal-attainment rate of LDL-C in patients from different departments of the same tertiary hospital. We only enrolled patients from one hospital, so as to achieve data more comparable under real-world conditions. At baseline, the major risk factors, including the prevalence of hypertension, smoking, gender, and levels of LDL- C, TC, TG, apo A1, and apo B, were comparable among the four groups (T2DM with or without CAD in either cardiology or endocrinology). However, there was a lower ratio of statin use in patients with T2DM with or without CAD and a lower ratio of patients undergoing percutaneous coronary intervention among the patients with T2DM and CAD in the department of endocrinology compared to the department of cardiology. This might reflect the current medical status that coronary intervention for CAD patients may be easier to implement in cardiology than in endocrinology. In this regard, physicians play an important role in patients' treatment selection and, therefore, more could be done in the practice of secondary prevention to increase the ratio of target-goal achievement.

At the 6-month follow-up, when compared to patients with T2DM in the department of endocrinology, the ratio of target-goal achievement of LDL-C among patients with T2DM in the department of cardiology was higher, indicating a significant improvement among these patients in cardiology when compared to baseline but not when compared to the patients in endocrinology. According to the new Chinese guidelines, the goal attainment rate was also higher among patients with T2DM and CAD in the department of cardiology than in endocrinology. However, with regard to ATP III 2004, the goal attainment rate was poor and similar among patients with T2DM and CAD from both departments, without any significant improvement compared to baseline, which was worse than that reported by Wu YF et al. [22]. In a study sample of 21,801 patients with CAD from one Veterans Affairs Hospitals Network, Virani SS et al. [24] found that LDL-C goal attainment was 80%, but optional LDL-C goal attainment was only 41%. In the present study, the low ratio of statin prescription did not increase even after the 6-month professional follow-up. This might partially explain the poor goal attainment rates in our study. Sub-optional LDL-C goal attainment can also be found in the study of Canadian patients based on the Canadian and American guidelines for lipid management [32].

To improve the sub-optimal therapy status in order to reduce long-term CVD risk, the measures taken should be considered with regard to the following factors: life style, statin dosage or combination therapy, patient risk, baseline LDL-C, gender, number of primary care visits, and physicians' education and working background, as these are all major factors affecting goal attainment [22], [24], [33]–[36], especially in regards to patients with any acute coronary syndrome eligible for elective, semi-urgent, or primary percutaneous coronary intervention [37]. First, awareness of guideline recommendations must be raised among physicians to increase optimal preventive measures. Second, regular nurse-led interventions, as well as telephone monitoring and internet-based self-education and management, should be strengthened to increase patients' therapeutically beneficial lifestyle changes, drug compliance, and monitoring [24], [34], [38], [39].

The present study has several strengths and limitations. First, because the study cohort was obtained from a single hospital, one may speculate that the subjects in our study were not representative of the general population. To this point, our hospital is a tertiary hospital located in a developed city in China, and it is reasonable to think the goal attainment rate surpasses the average level of the whole country. Second, our study preserves its validity because it benefits from a well-designed study protocol that has been carried out using well-established methods, with the advantage that all the participants underwent CAG to make the diagnosis of CAD accurate. Additionally, it is generally accepted that patients with T2DM and CAD belong to very high-risk group in practice, among patients with diagnosed T2DM, we only enrolled patients with stable CAD to minimize the influence of unstable disease condition on the lipid levels. However, we did not evaluate the attainment of non-HDL-C goals in this study, which should not be ignored in the control of total cardiovascular risk in real-world settings [23], [24].

## Conclusions

For the first time, we have reported the differential and low statin usage and LDL-C target-goal achievement in T2DM patients with or without stable CAD between the cardiology and endocrinology departments at a tertiary hospital in Southeast of China. The clinical relevance of the present findings lies in the fact that a large gap still exists between reality and the recommended goals in the guidelines. Further studies are needed to investigate the causes underlying this status, and active interventions to improve this sub-optimal status, especially in these high and very high-risk populations, should be pursued.
